# Müllerianosis of the Urinary Bladder: A Rare Condition Which Mimics Neoplasia

**DOI:** 10.7759/cureus.15147

**Published:** 2021-05-21

**Authors:** Somshukla Ghosh, Fikret Onol, Jonathan Vasquez, Jignesh Parikh

**Affiliations:** 1 Internal Medicine, University of Central Florida-HCA Healthcare Graduate Medical Education, Greater Orlando, USA; 2 Urology, Orlando Veterans Affairs Medical Center, Orlando, USA; 3 Pathology, Orlando Veterans Affairs Medical Center, Orlando, USA

**Keywords:** müllerianosis of urinary bladder, pseudoneoplastic bladder lesion, urinary bladder malignancy, hematuria, pelvic pain

## Abstract

Müllerianosis of the urinary bladder is an extremely rare, benign condition defined by the presence of at least two of the three müllerian-derived components (endosalpinx, endometrium, and endocervix) in the lamina propria and muscularis propria of the urinary bladder. It frequently mimics neoplastic condition, either malignant or benign. Here, we present a case of cystic müllerianosis of urinary bladder, which was clinically thought to be a urinary bladder neoplasm.

## Introduction

Müllerianosis of the urinary bladder is a benign condition characterized histopathologically by the presence of at least two of the three müllerian-derived components (endosalpinx, endometrium, and endocervix) in the lamina propria and muscularis propria of the urinary bladder [1]. It is an extremely rare condition, mostly affecting women in the child-bearing age group [2]. Symptoms include abnormal uterine bleeding, hematuria, dysuria, pelvic pain, and suprapubic pain. Radiologically, müllerianosis can be seen as a polypoid mass by ultrasound, computed tomogramy (CT), or magnetic resonance imaging (MRI). However, a preferred diagnostic imaging modality has not been identified because of the small number of reported patients with this condition. Differential diagnosis encompasses neoplastic conditions including invasive cancer of the genitourinary tract, and hence, accurate diagnosis is crucial for appropriate treatment. The potential for urinary bladder müllerianosis to transform into malignant neoplasia and recommended treatment modalities are also unclear.

## Case presentation

A 31-year-old nulliparous woman presented with the complaints of menorrhagia and pelvic pain for seven months. She also complained of pain during micturition and increased urinary frequency during her menstrual cycles for the same duration. Contrast-enhanced CT scan of the abdomen and pelvis showed multiple uterine fibroids with both subserosal and submucosal components. To relieve her bulk symptoms, she underwent laparoscopic total hysterectomy with bilateral salpingectomy. During the laparoscopic surgery, a 2-3 cm nodule was visualized above the urinary bladder trigone. The nodule was hard and did not extend through the bladder serosa. Of note, the CT scan prior to the surgery did not show this lesion.

**Figure 1:**
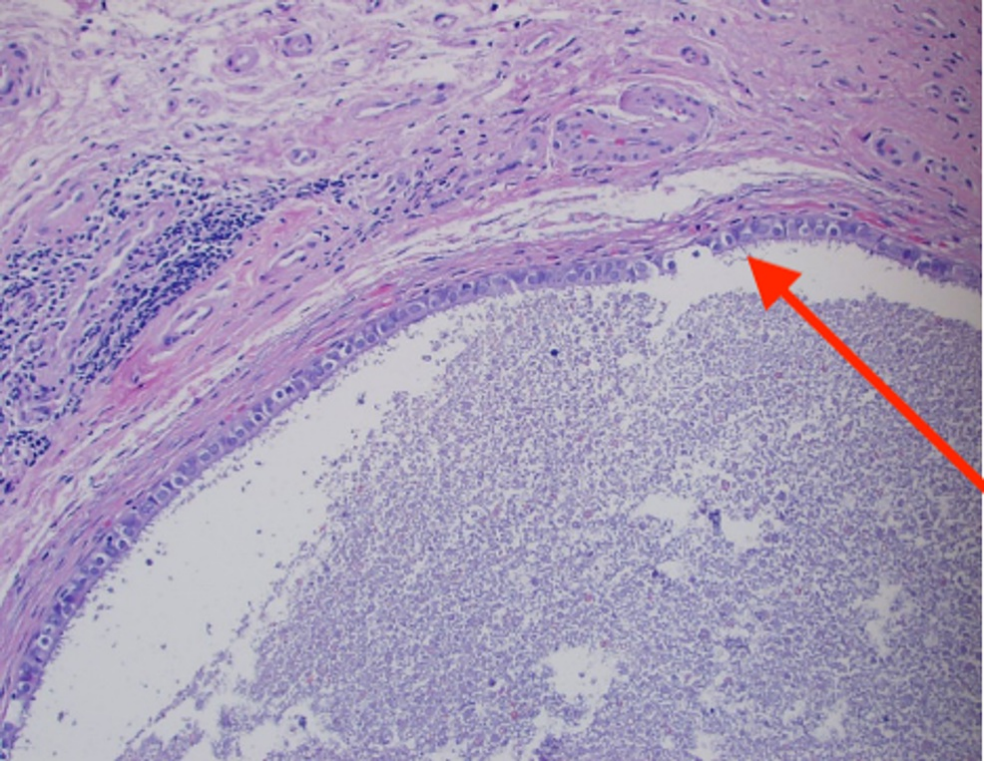
Hematoxylin and eosin-stained section showing ciliated epithelium-lined cyst.

**Figure 2:**
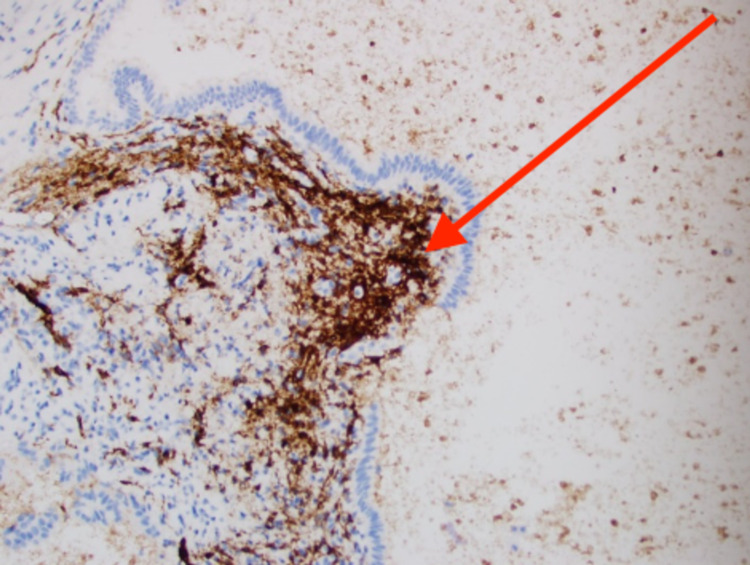
Pericystic stromal tissue focally positive for CD10 immunostain.

**Figure 3:**
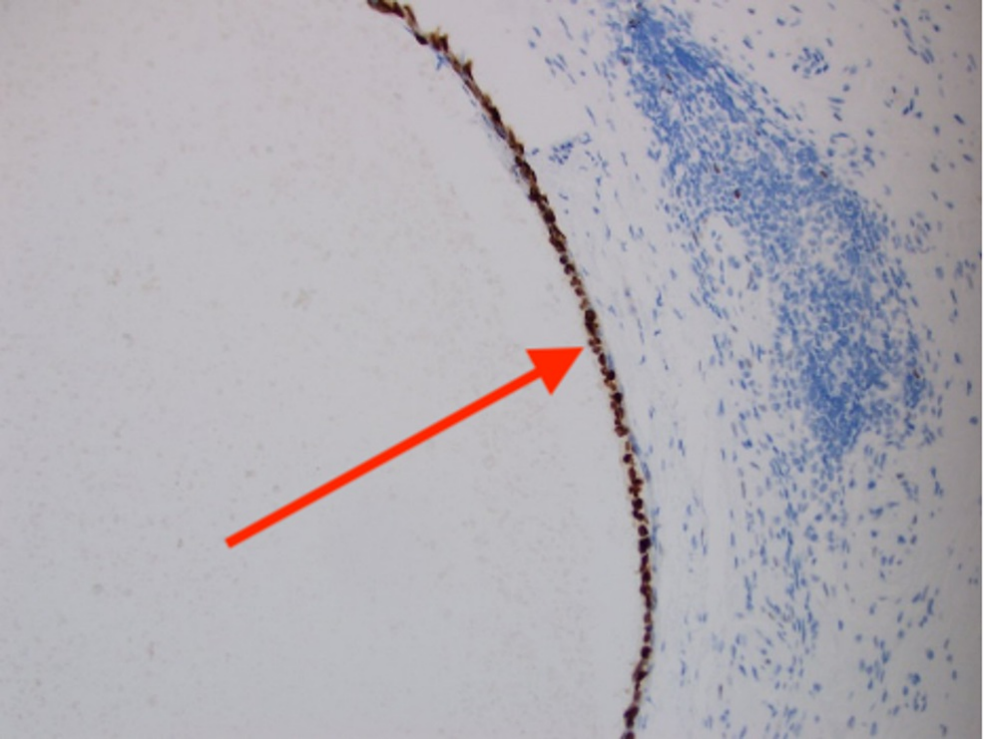
Cyst lining cells positive for immunostains for ER. ER: estrogen receptor

**Figure 4:**
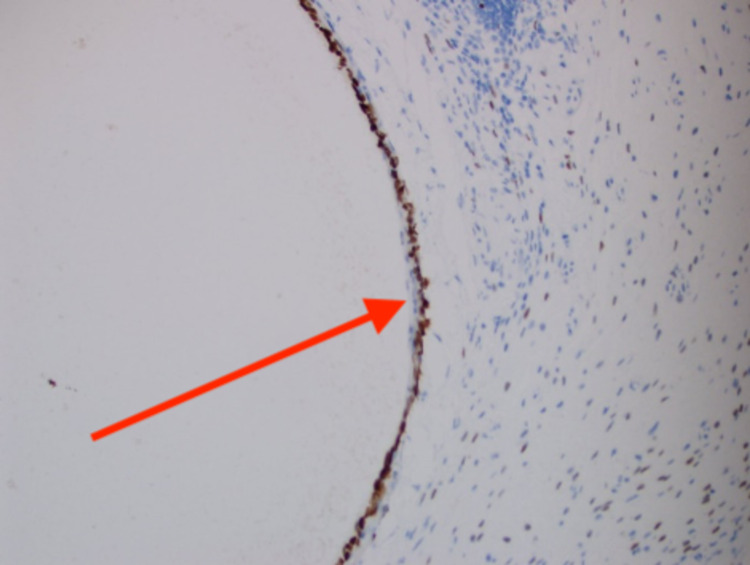
Cyst lining cells positive for immunostains for PR. PR: progesterone receptor

For further evaluation, the patient underwent an elective cystoscopy which revealed a nodular lesion with calcification on the posterior wall of the urinary bladder, which was highly suspicious for a neoplasm. The patient underwent a transurethral resection of this urinary bladder nodule. Histopathological examination showed a cyst within the urinary bladder wall, lined by tubal-type epithelium and endometrial-like pericystic stroma (Figure 1). CD10 staining (Figure 2) was focally positive within pericysticstromal tissue, and estrogen receptor (ER) (Figure 3) and progesterone receptor (PR) (Figure 4) immunostains were positive within the cyst lining cells. The morphology and immunohistochemical stains supported the diagnosis of cystic müllerianosis. Her urinary symptoms resolved after its resection, and she did not show any signs of recurrence during a follow-up of 12 months.

## Discussion

Müllerianosis of the urinary tract is an extremely rare condition with less than 40 cases reported to date [2-14]. It is a benign condition and the most common sites reported are the trigone or posterior wall of the urinary bladder, or the lower ureter or the ureterovesical junction. So far, all the cases reported have been in women, mostly in the child-bearing age group. There have been only three reported cases so far in post-menopausal women [3,13,15]. This condition is characterized histopathologically by the presence of at least two of the three müllerian-derived components (endosalpinx, endometrium, and endocervix) in the lamina propria and muscularis propria of the urinary bladder [1]. However, there are also cases reported where the bladder lesion, on histopathology, has shown tubal-type epithelium only [16]. To our knowledge, only five such cases have been documented in the English language literature [16-19], and this entity is called endosalpingosis.

Symptoms include abnormal uterine bleeding, hematuria, dysuria, pelvic pain, and suprapubic pain, which may or may not be associated with menstruation. Patients with ureteric lesions often had symptoms suggestive of ureteric obstruction or hematuria. The symptoms of our patient were associated with menstruation.

Both implantative and metaplastic origins have been suggested as pathogenesis of these lesions [3,18]. In nine of the cases reported previously, the patients had prior history of pelvic surgery or caesarian section, thus supporting the implantative pathology. However, our patient was nulliparous and did not have any history of pelvic surgery; hence, the likelihood of metaplastic origin is higher.

Ultrasound, MRI, or urinary cytology showing epithelial aggregates with papillary configurations have been useful for the diagnosis. Radiologically, müllerianosis can be seen as a polypoid mass on ultrasound, CT scan, or MRI. A preferred diagnostic imaging modality has not been identified because of such a small number of patients that have been reported with this condition. Interestingly, CT scan of the abdomen and pelvis did not reveal any urinary bladder nodule in our patient, and it was only seen on cystoscopy. There have been two cases reported in which the diagnosis was suggested by urine cytopathology which showed endometrium-like cells [12].

Differential diagnosis of müllerianosis of the bladder includes cystitis glandularis, cystitis cystica, bladder adenocarcinoma, carcinoma of the urachus, and neoplastic lesions, including cervical cancer invading into the bladder wall. Definitive diagnosis is by histopathologic evaluation after cystoscopic removal of the lesion.

Treatment of müllerianosis is controversial, and modalities include pharmacotherapy with gonadotropin-releasing hormone (GnRH) agonist or with surgical resection of the lesion. Two cases have been reported where treatment with GnRH resulted in resolution of symptoms; however, the size of the lesion continued to persist [3,11]. Because of the concern for its potential malignant transformation [7], resection seems to be the preferred treatment.

Despite the benign nature of bladder müllerianosis, some reports have described malignant transformation of the müllerianosis into bladder endometroid carcinoma [7]. Due to very limited number of known cases, the potential for bladder müllerianosis to transform into malignant neoplasia is unknown. However, patients should be followed up with cystoscopy even after successful surgical removal of the lesion. They should also be monitored for recurrence of symptoms. There is no recommended time interval for repeat cystoscopies due to the very small number of cases. Our patient has been doing well with complete resolution of all her symptoms at one-year follow-up after the removal of the mass. She is yet to have a repeat cystoscopy.

In summary, müllerianosis of the urinary bladder closely mimics a urinary bladder neoplastic lesion in terms of clinical presentation, cytology, and cystoscopic findings [11,19]. Accurate diagnosis is crucial as correct diagnosis will prevent the patient from undergoing radical cystectomy which is the standard treatment for invasive urinary bladder malignancy.

## Conclusions

Müllerianosis is a rare condition that can be considered to be a pseudoneoplastic bladder lesion potentially responsible for a false diagnosis of malignancy. Due to its rarity, definitive guidelines for management are lacking. Recommended imaging modality, probability of malignant transformation, and time interval for surveillance are few of the many areas that need further exploration. We report this case to add to the general knowledge about müllerianosis.
